# Adequacy of antenatal care services utilisation and its effect on anaemia in pregnancy

**DOI:** 10.1017/jns.2022.80

**Published:** 2022-09-21

**Authors:** Ferguson Saapiire, Richard Dogoli, Saaka Mahama

**Affiliations:** 1 Ministry of Health, St. Joseph Nursing Training College, Jirapa, Ghana; 2 Jhpiego Ghana, PMB 18, Legon Accra, Ghana; 3 Department of Nutritional Sciences, University for Development Studies, Tamale, Ghana

**Keywords:** Anaemia, Antenatal care utilisation, Ghana, Pregnancy, Wa municipality

## Abstract

Anaemia in pregnancy remains a critical public health concern in many countries including Ghana and it poses severe consequences in the short to long-term for women and their unborn babies. Although antenatal care (ANC) is largely provided for pregnant women, the extent its utilisation protects against anaemia in pregnancy remains largely understudied. The study assessed the adequacy of ANC services utilisation and its effect on anaemia among pregnant women in the Wa Municipality of Ghana. A facility-based cross-sectional survey was conducted. Probability proportionate to size sampling and systematic random sampling were used to select the facilities and 353 respondents. While 80⋅2 % of the pregnant women reported having received a sufficient number of ANC services provided, the prevalence of the overall ANC adequacy was only 44⋅2 %. After adjusting for potential confounders, pregnant women who could not achieve adequate ANC attendance were 2⋅3 times more likely to be anaemic in the third trimester of gestation AOR = 2⋅26 (95 % CI 1⋅05, 4⋅89), compared to their counterparts who maintained adequate ANC attendance. Adequate ANC attendance was a consistent and significant predictor of anaemia in pregnancy in the third trimester. Health and nutrition education on the need for early initiation of ANC attendance and support for the consumption of diversified diets are two possible interventions that can help contain anaemia in pregnancy.

## Introduction

Maternal anaemia remains a serious global public health threat that affects over 38 % of pregnant women globally with 18 % in high-income countries and 35–75 % in low- and middle-income countries^(1)^. The World Health Organization (WHO) defines pregnancy anaemia as those expectant mothers with haemogloblin (Hb) concentration lesser than 11⋅0 g per deciliter when at sea level or when the total volume ratios of the red blood cells to blood is less than 33⋅0 %, irrespective of gestation^(2,3)^.

According to the Ghana Demographic Health Survey, anaemia prevalence among pregnant women in Ghana was 45 % which has not seen much improvement over the years^(4)^. In the Wa Municipality of Ghana where the present study was conducted, the prevalence of anaemia at 36 weeks gestation in 2016 was 35⋅4 %^(5)^.

Anaemia in pregnancy account for 20 % of maternal mortality worldwide^(1)^ and it is a major nutritional disorder that poses severe consequences in the short to long-term for women and their unborn babies^(6)^. It has been established from several studies that anaemic expectant women stand a higher chance of having premature births and delivery of low-birth-weight babies and preterm delivery^(7–9)^.

The major determinant of pregnancy anaemia worldwide is known to be iron deficiency^(10)^. However, poor antenatal care (ANC) quality and nutrition might also possibly explain the observed high prevalence of anaemia during pregnancy^(11)^ especially in developing countries^(12)^. There has been an increase in the utilisation of antenatal services in low- and middle-income countries (LMICs) following the WHO recommended focused antenatal care (FANC) practice. FANC involves client-centered individualised care, disease detection and care by a skilled provider^(13)^.

Despite the general increase in the frequency of ANC attendance, timely initiation remains problematic in many settings. In Northern Ghana, a study reported that although 75⋅0 % of pregnant women made at least four ANC visits in their last pregnancy, late initiation of ANC services was widespread^(14)^. Poor quality and inadequate ANC visits during pregnancy can increase the risk of anaemia. This is because, timely initiation and regular ANC visits give health personnel an opportunity to provide a variety of services including prophylaxis and micronutrient supplementation^(15,16)^, which are preventative measures against anaemia. It has also been established that a combination of interventions may yield a stronger effect compared to one type of intervention^(17)^. ANC, therefore, presents a unique opportunity for the prevention and management of concurrent diseases through integrated service delivery^(18)^.

The persistent high prevalence of anaemia in many settings suggests that the most important risk factors have not been addressed yet and so many healthcare systems continue to seek for appropriate and effective measures to manage this situation. One of such measures is the promotion of ANC which serves as a platform for the delivery of protective health and nutrition interventions during pregnancy. Even though timely initiation and frequent ANC attendance as recommended by the WHO are the major strategies available to improve maternal health outcomes, limited evidence exists on the independent contribution of adequate ANC in protecting against pregnancy anaemia. The study assessed the adequacy of ANC services utilisation and its effect on anaemia among pregnant women in the Wa Municipality of Ghana.

## Materials and methods

### Study area

Wa Municipality is sited in the northern savannah part of Ghana between Latitudes 8°30″–10° N and Longitude 0°30″–2°30″W but lies in the Southwestern part of the Region between Longitudes 9°32″W and 10°20″W and Latitudes 1°40″N and 2°45″N. The Wa Municipality is subdivided into six (6) sub-municipals with a total of twenty-six government health facilities including community-based health planning and services (CHPS) and four private facilities^(19)^. However, data from 2017 showed the total number of health facilities in the Municipality to be forty-five^(5)^.

The Wa Municipal has 132 communities with one paramountcy, four area councils and one urban council^(20)^. The municipality has an estimated total land area of 579⋅86 km2 and a projected population of 107 214 comprising of 52 996 males and 54 218 females.

### Study design, study population and sampling

A facility-based cross-sectional survey was conducted. The study population comprised all women who had attended ANC in the municipality, delivered within the past 12 months preceding the study in a health facility and possessed a maternal health records booklet for the index pregnancy.

The minimum sample size was calculated using single population proportion formula:
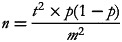
where *n* is the required sample size, *t* is the confidence level at 95 %, *P* represents the population proportion of anaemia (35⋅6 %)^(21)^ and *m* is the margin of error at 5 %. Considering a 2 % contingency to take care of incomplete/damaged questionnaires, a total of 353 mothers were required for the study.

Health facilities for the study were selected from all six sub-districts in the Wa Municipality using probability proportionate to size sampling technique based on the total ANC registrants.

The study participants were selected using systematic random sampling to draw respondents from ANC registers. Women were excluded if they were referred from other district's ANC facilities but came to deliver in the Wa Municipality or attended ANC elsewhere for the most part of their pregnancy. Women without maternal health records or booklet for their index pregnancy were also excluded.

### Data collection methods

A pre-tested structured questionnaire was used to collect information on socio-demographic characteristics, maternal behaviours and health status during last pregnancy, household wealth index, gravidity and parity. Information extracted from the ANC booklet included timing of ANC initiation, gestational age and Hb concentration levels.

Data for the present study were collected in 2019 by trained nurses and midwives with a minimum qualification of Diploma. ANC registers were major sources of data. Permission was granted by the health authorities of the institutions to have access to the ANC registers where data were extracted.

### Measurement of independent and dependent variables

The main outcome (dependent) variable of interest was anaemia in the third trimester of pregnancy. The WHO's definition and categorisation were applied whereby women with haemoglobin (Hb) concentration levels more than or equal to 11 and <11 g/dl were classified as ‘not anaemic’ and ‘anaemic’, respectively^(2)^.

The Hb levels were determined using a portable HemoCue301 photometer. Trained laboratory technicians drew capillary blood samples from the finger prick with a lancet after taking all aseptic precautions. The first drop of blood was wiped away using alcohol sterile wipes, and the next drop was placed into the HemoCue cuvette for immediate testing of Hb.

The main exposure variable was the adequacy of ANC utilisation which was measured using a modified version of the adequacy of prenatal care utilisation (APNCU) index^(22)^. The APNCU index is used for precise and comprehensive measurement of prenatal care^(23)^. To be considered having adequate ANC attendance, a mother must have initiated ANC before gestational age (GA) week 12, and subsequent visits must be attended at recommended intervals throughout the pregnancy. The ultrasound technology was used to determine gestational age on first booking at the ANC. In the absence of ultrasound facility, gestational age was assessed using the last menstrual period (LMP) approach.

The WHO recommends at least four antenatal visits to healthcare facility during pregnancy^(20)^. Adequacy of ANC attendance in this study was therefore measured as having made the first visit in the first trimester of pregnancy and attended ANC at least four times during pregnancy.

To assess ANC service content, participants were asked about the basic ANC services received as recommended by the WHO for all pregnant women at the first visit to ANC clinics^(24)^.

The content of ANC services received during antenatal period was assessed included height and weight measurements, blood pressure and blood sugar, deworming, toxoid immunisation, malaria testing and treatment, health and nutrition education, blood and urine testing, iron and folic acid supplementation. A score of ‘1’ was assigned to receiving any of the services and ‘0’ for non-receipt. The total score for each respondent was categorised as low, if that score was below the median score and high, if it was at least the median score.

Another important exposure variable measured was a composite indicator that reflects an overall ANC adequacy. This was constructed using the three ANC utilisation indicators (that is, first ANC visit made during first trimester, making at least four ANC visits and receipt of adequate ANC core services^(^^24)^. Thus, a woman was classified as having adequate overall ANC, if the woman had attended prenatal care early plus enough visits and sufficient services; otherwise, she was classified as having inadequate ANC.

Based on the literature, the other potential predictor/covariate variables included were socio-demographic factors such as age, maternal education, occupation, marital status, religion, whether the women received health and nutrition education, child vaccinations and immunisations, the number of tetanus toxoid (TT) and sulphadoxine pyrimethamine (SP) received, household wealth index, as well as maternal dietary intake. Details of some of these variables are given below:

The minimum dietary diversity for women (MDD-W) was used as measure of overall dietary quality since it has been shown to indicate adequate nutrient intake and can be used as a proxy indicator for measuring nutrient adequacy among pregnant women^(25,26)^. The dietary assessment was made by asking the women to recall all foods and drinks consumed in the past 24 h prior to the study^(26)^.

The women's dietary diversity scores (WDDSs) were calculated by adding the values of all the food groups consumed by each participant. The ten food groups used to calculate WDDS were starchy staple foods, beans, peas, nuts, seeds, dairy, flesh foods, eggs, vitamin A-rich dark green leafy vegetables, other vitamin A-rich vegetables and fruits, other vegetables, and other fruits.

The consumption of a food item from any of the groups was assigned a score of ‘1’ and a score of ‘0’ if the food was not consumed. The WDDSs were used to categorise the women into high (WDDS ≥ 5) and low (WDDS < 5).

Also assessed was the household wealth index, which is a proxy indicator for socio-economic status (SES) of households. The principal component analysis (PCA) was used to quantify it from information collected on household assets and housing quality (floor, walls and roof material), source of drinking water, type of toilet facility, the presence of electricity, type of cooking fuel and ownership of modern household durable goods and livestock (e.g. bicycle, television, radio, motorcycle, sewing machine, telephone, cars, refrigerator, mattress, bed, computer and mobile phone)^(27–30)^.

### Statistical data analysis

The data were cleaned and coded for analysis using the Statistical Package for Social Science (SPSS) version 22 (SPSS Inc, Chicago). Data were cleaned by running preliminary frequencies of all the variables to check for entry inaccuracies. All incorrectly coded data were double-checked with the questionnaire after which all wrong entries were corrected.

Bivariate analysis was performed using Chi-square test of independence to assess the association between the dependent variable anaemia, and categorical independent variables. The variables in the bivariate analysis with *P* < 0⋅10 were included in the multivariable binary logistic regression to control for possible confounding and the independent effect of each independent variable on the outcome variable. Forward stepwise LR (likely hood ratio) method was used for entering variables.

The adjusted odds ratio (AOR) and 95 % confidence intervals were used to assess the strength of association at *P*-value < 0⋅05.

Multicollinearity was checked using variance inflation factor (VIF) and no collinearity existed between the independent variables.

### Ethics consideration

The study protocol was approved by the School of Allied Health Sciences, University for Development Studies, Ghana. Ethical approval was obtained from the Kwame Nkrumah University of Science and Technology ethics committee (Reference no. CHRPE/AP/472/21). Permission was also granted by the Regional Health Directorate and District Director for Health Services of Wa Municipal to carry out the survey. Informed consent was obtained from the study participants prior to data collection. Confidentiality and anonymity of the study participants was also maintained by using identity numbers on the questionnaires other than participant's names.

## Results

### Socio-demographic characteristics of respondents

Out of a total of 353 study respondents, an overwhelming majority (85⋅0 %) were Waala/Dagaaba tribe. The predominant religion was Islam (84⋅4 %). In terms of marital status, almost all the respondents (91⋅2 %) were duly married. More than a third of the study participants (36⋅3 %) never had any formal education while (23⋅8 %) were able to attain at least a second cycle education (senior high and tertiary levels). It was also observed that about 38⋅0 % of the respondents were unemployed and a few on apprenticeship in various vocations including hairdressing, dressmaking or weaving. Aside 8⋅2 % that were engaged as government/private employees, 53⋅8 % were into self-occupation either as a farmer or as a trader in cooking/brewing and selling, buying and selling of commodities. With regard to household wealth index, 56⋅1 % of the respondents were above the median (Table 1).

**Table 1.** Socio-demographic characteristics of the respondents (*N* = 353)

Characteristic	Frequency (*n*)	Percentage
Age of mother (years)		
Under 20	28	7⋅9
20–34	273	77⋅3
35+	52	14⋅7
Marital status		
Not married	31	8⋅8
Married	322	91⋅2
Ethnicity		
Wala/Dagaaba	300	85⋅0
Sissala	16	4⋅5
Dagomba	11	3⋅1
Others	26	7⋅4
Religion		
Muslim	298	84⋅4
Christian	55	15⋅6
Maternal formal education level		
None	128	36⋅3
Low (Primary and JHS)	141	39⋅9
High (At least SHS)	84	23⋅8
Maternal occupation		
No occupation	134	38⋅0
Self-employed	190	53⋅8
Government/Private employee	29	8⋅2
Household wealth index		
Low	155	43⋅9
High	198	56⋅1

### Content of ANC services received during antenatal period

In the study sample, majority (60⋅3 %) had adequate requisite information. Most of the women (73⋅1 %) received less than two doses of tetanus toxoid (TT) for their most recent pregnancy. For sulphadoxine pyrimethamine (SP) intake, a vast majority of the women (82⋅4 %) had received at least two doses as stipulated by WHO for the minimum protection prophylaxis against malaria (Table 2).

**Table 2.** Content of ANC services received during antenatal period

Variable	*N* (%)
Weight checked at least two times	350 (99⋅2)
Height taken on first visit	334 (94⋅6)
Blood pressure taken at least three times	328 (92⋅9)
Urine examination performed at least once	329 (93⋅2)
Blood sample examination performed at least once	344 (97⋅5)
Received health and nutrition talk at least four times on possible danger signs/complications of pregnancy	130 (36⋅8)
Received TT injection at least once	323 (91⋅5)
Received iron supplementation monthly	339 (96⋅0)
Measurement of fundal height, foetal heartbeat and foetal movement count monthly	348 (98⋅6)
Received information on childhood diseases	161 (45⋅6)
Received information on nutrition of mothers and children	274 (77⋅6)
Received information on breast-feeding	271 (76⋅8)
Received information on antenatal and delivery care	296 (83⋅9)
Received information on vaccinations and immunisations	302 (85⋅6)
Received information on family planning	237 (67⋅1)
Number that received high average score for information on essential services	213 (60⋅3)
Number that received TT 2+	95 (26⋅9)
Score for SP intake	
Inadequate (<2+ doses)	62 (17⋅6)
Adequate (2+ doses)	291 (82⋅4)

### Utilisation of antenatal care services

Adequacy of ANC attendance as measured by at least four ANC visits during pregnancy and making the first visit in the first trimester of pregnancy was low at 49⋅9 %.

More than half (51⋅3 %) of the ANC attendees-initiated ANC attendance in the first trimester of pregnancy and 80⋅7 % of the women had four or more ANC visits during the last pregnancy as recommended by the WHO. While 80⋅2 % of the pregnant women reported having received a sufficient number of ANC services provided, the prevalence of the overall ANC adequacy was only 44⋅2 % (Table 3).

**Table 3.** Utilisation of antenatal care services (*N* = 353)

Variables	Frequency (*n*)	Percent (%)
Frequency of ANC visits		
<4	68	19⋅3
4+	285	80⋅7
Gestational age at first ANC attendance		
First trimester (0–3 months)	181	51⋅3
Second trimester (4–6 months)	140	39⋅7
Third trimester (7–9 months)	32	9⋅1
Adequacy of ANC utilisation		
Inadequate	177	50⋅1
Adequate	176	49⋅9
Classification of content of ANC services received		
Low (<median of 8)	70	19⋅8
High (At least 8)	283	80⋅2
Overall ANC adequacy (meeting both adequacy of attendance and content of ANC)	156	44⋅2

### Relationship between anaemia in the third trimester of pregnancy and selected factors

Table 4 shows the relationship between selected factors including the three main components of adequacy of ANC services utilisation and anaemia (Hb less than 11 g/dl) at 36 weeks of gestation. Adequacy of ANC attendance negatively associated with the prevalence of anaemia but the number of ANC services received (content) did not associate significantly with anaemia in the third trimester.

**Table 4.** Relationship between anaemia in the third trimester of pregnancy and selected factors (bivariate analysis)

Predictor	*N*	Outcome		
		Anaemia at 36 weeks gestation *n* (%)		Test statistic
Adequacy of ANC attendance (First ANC contact in first trimester and making at least 4 visits)		Non anaemic	Anaemic	
No	171	76 (44⋅4)	95 (55⋅6)	*χ^2^* = 19⋅4 *P* < 0⋅001
Yes	172	117 (68⋅0)	55 (32⋅0)	
Classification of content of ANC services received				
Low (<median of 8)	65	36 (55⋅4)	29 (44⋅6)	*χ^2^* = 0⋅3 *P* = 0⋅9
High (At least median score of 8)	278	157 (56⋅5)	121 (43⋅5)	
Overall ANC adequacy (meeting both adequacy of attendance and content of ANC basic services)				
No	189	90 (47⋅6)	99 (52⋅4)	χ*^2^* = 12⋅8 *P* < 0⋅001
Yes	154	103 (66⋅9)	51 (33⋅1)	
Household wealth index				
Low (Below median score)	149	75 (50⋅3)	74 (49⋅7)	χ*^2^* = 3⋅8 *P* = 0⋅05
High (At least median score)	194	118 (60⋅8)	76 (39⋅2)	
Classification of 7-day DDS				
Low (<median score)	161	95 (59⋅0)	66 (41⋅0)	χ*^2^* = 0⋅9 *P* = 0⋅3
High (At least median score)	182	98 (53⋅8)	84 (46⋅2)	
Gestation at first ANC visit				
First trimester	176	120 (68⋅2)	56 (31⋅8)	χ*^2^* = 20⋅9 *P* < 0⋅001
Beyond first trimester	167	73 (43⋅7)	94 (56⋅3)	
Frequency of ANC visits				
Less than 4 visits	64	27 (42⋅2)	37 (57⋅8)	χ*^2^* = 6⋅3 *P* = 0⋅01
At least 4 visits	279	166 (59⋅5)	113 (40⋅5)	

### Predictors of anaemia in the third trimester of pregnancy

After adjusting for potential confounders, adequacy of ANC attendance was a consistent significant predictor of anaemia in the third trimester (Table 5). Pregnant women who could not achieve adequate ANC attendance were 2⋅3 times more likely to be anaemic in the third trimester of pregnancy AOR = 2⋅26 (95 % CI 1⋅05, 4⋅89), compared with their counterparts who maintained adequate ANC attendance.

**Table 5.** Predictors of anaemia at 36 weeks of gestation among pregnant women (multiple logistics regression)

	Wald	Sig.	Adjusted odds ratio (AOR)	AOR (95 % CI)	
				Lower	Upper
Low BMI	15⋅905	<0⋅001	1⋅16	1⋅08	1⋅25
Women's dietary diversity score (WDDS)	7⋅541	0⋅006	0⋅44	0⋅25	0⋅79
Gestational weight gain	10⋅335	0⋅001	0⋅88	0⋅08	0⋅95
Rural residence	6⋅481	0⋅011	2⋅09	1⋅19	3⋅69
Inadequate ANC attendance	4⋅289	0⋅038	2⋅26	1⋅05	4⋅89
Constant	12⋅364	<0⋅001	0⋅03		

The potential confounding variables adjusted for included age of mother, gravidity, maternal educational level, early pregnancy BMI, weight gain during pregnancy, place of residence, the number of ANC services received (content), household wealth index, women's dietary diversity score (WDDS), whether anaemic in early pregnancy and gestational age at first ANC visit.

Lower BMI in early pregnancy was associated with 1⋅2-fold increase in the odds of anaemia, AOR = 1⋅16 (95 % CI 1⋅08, 1⋅25) whereas a unit increase in gestational weight gain led to 12 % protection against anaemia AOR = 0⋅88 (95 % CI 0⋅08, 0⋅95). A unit increase in women's dietary diversity score (WDDS) was associated with 56 % protection against anaemia AOR = 0⋅44 (95 % CI 0⋅25, 0⋅79). Women resident in the rural areas were 2⋅1 times more likely of being anaemic in the third trimester, compared with their counterparts in the urban settings AOR = 2⋅09 (95 % CI 1⋅19, 3⋅69).

The set of factors accounted for 23⋅7 % of the variance in anaemia at 36 weeks gestation (Nagelkerke R square = 0⋅237).

## Discussion

The study assessed the adequacy of ANC services utilisation and its effect on anaemia among pregnant women in Ghana. The key finding was that adequacy of ANC attendance was low at 49⋅9 %. Pregnant women who could not achieve adequate ANC attendance were 2⋅3 times more likely to be anaemic in the third trimester of pregnancy compared with their counterparts who maintained adequate ANC attendance.

### Adequacy of prenatal care services utilisation

The adequacy of ANC attendance as measured by at least four ANC visits during pregnancy and making the first visit in the first trimester of pregnancy was low at 49⋅9 %. Though more than 80 % of the pregnant women had four or more ANC visits during the last pregnancy, only 51⋅3 % of them initiated ANC attendance in the first trimester of pregnancy. At least 80 % of the women received sufficient ANC services but the overall ANC adequacy was only 44⋅2 %. As pointed out by Sumankuuro and colleagues, the barriers for adequate utilisation of ANC services include long distance to ANC facilities, transport difficulties, cultural influence and social reasons^(31)^. Several other studies have reported that in some cases, women might just not feel the need to seek professional care when there is nothing wrong with their pregnancy^(32–34)^.

In order to derive maximum benefits from ANC services, it is critically important that every pregnant woman makes the first ANC visit during the first 3 months of pregnancy (timeliness), achieving a minimum of four, most recently at least eight contacts for ANC (frequency) and adequate services (ANC content)^(35,36)^. Generally, the content, timing and frequency of ANC services are inadequate in low- and middle-income countries (LMICs)^(37–39)^. In our sample, the late initiation of ANC attendance appears to be a major limitation to attaining the overall ANC adequacy. The focused ANC model recommends a minimum of four prenatal care visits and content of the packages that comprise physical examinations, laboratory investigations, preventive procedures and counselling on signs of pregnancy complications and measures to be taken by the mother^(40)^. As observed in our study sample, the timely initiation of ANC should be a focus to reap the desired effect of ANC services since the other pre-requisites were met by most pregnant women.

### Relationship between ANC services utilisation and pregnancy anaemia in the third trimester

After adjusting for potential confounders, inadequate ANC attendance positively associated with the prevalence of anaemia in the third trimesters. This finding is consistent with some studies^(41,42)^. This finding may be explained by the fact that women who initiate ANC late in either the second or third trimester will definitely miss many of the interventions and services routinely offered to pregnant women at ANC clinics to prevent anaemia in pregnancy. Some of these routine preventive measures include iron-folic acid (IFA) supplementation, provision of long-lasting insecticide nets (LLINs) and sulphadoxine pyrimethamine (SP) dosing, as well as laboratory investigations to diagnose early anaemia in pregnancy. Indeed, early initiation of ANC is critical in preventing anaemia in pregnancy and other pregnancy outcomes. In particular, mothers with early signs of pregnancy anaemia who seek ANC services in the first trimester will be timely managed^(43,44)^. This points to the urgent need for both health facility and community-based interventions to educate women and community members on the importance of early ANC initiation.

From our results, both timing and frequency of ANC attendance associated negatively with anaemia but the sufficiency of ANC services received did not. The lack of association between ANC services and anaemia may possibly be due to the limited variability of services among the study participants. When the content of ANC services provided to groups of women does not vary very much, it is the adequacy of ANC attendance that makes the difference. A woman who timely and frequently utilise ANC will benefit more from whatever quality services that are provided.

Other significant predictors of anaemia in the third trimester were early pregnancy BMI, weight gain during pregnancy, dietary diversity score and rural residence. These factors should also be considered when planning for interventions to improve the anaemia in pregnant women. For example, nutrition education on the consumption of diversified and iron-enriched foods during pregnancy should routinely be delivered in all ANC clinics.

Pregnant women who reported of high dietary diversity scores had 56 % protection against anaemia, compared with those with a low dietary diversity score. This finding is consistent with other studies^(45,46)^. Pregnant women on diversified diets are more likely to meet their micronutrients requirements such as iron, vitamin A and vitamin C all of which are necessary for blood formation. Improved consumption of iron-rich foods before and during pregnancy can therefore prevent anaemia. Unfortunately, foods of pregnant women in less developed countries are mostly carbohydrate-based with very little of micronutrient-rich vegetables, fruits and animal source foods^(47,48)^. The literature has it that the prevalence of anaemia is highest in the third trimester of pregnancy^(46,49–51)^ because of the high nutrient requirement for the growing foetus which will reduce the maternal iron reserves. This problem can be minimised through dietary diversification.

Pregnant women living in a rural area were two times more likely to be anaemic than those who live in urban areas. This is consistent with a number of studies^(45,52)^ and the relative high prevalence of anaemia in the rural areas might be due to several factors including socio-economic deprivations, cultural differences and a lack of information about adequate nutrition^(53)^.

In our study sample, lower BMI in early pregnancy was positively associated with increased risk of anaemia in the third trimester. This finding is consistent with a study that assessed the association between early pregnancy BMI and anaemia at first ANC visit in Indonesian and Ghanaian women which showed that higher BMI in early pregnancy associated with higher haemoglobin levels at antenatal booking and with a reduced risk of anaemia^(54)^.

Several other studies have earlier reported that low BMI in pre- or early pregnancy increases the risk of anaemia^(55–58)^. Low early pregnancy BMI may be due to poor diets that lack various micronutrients that are essential for haematopoiesis^(59)^. Additionally, low BMI could result from chronic illness or parasitic infections such as malaria, which can cause anaemia^(55,60)^.

## Conclusion

While 80⋅2 % of the pregnant women reported having received a sufficient number of ANC services provided, the prevalence of the overall ANC adequacy was only 44⋅2 %. After adjusting for potential confounders, adequate ANC attendance was a consistent and significant predictor of anaemia in pregnancy in the third trimester. Health and nutrition education on the need for early initiation of ANC attendance and support for the consumption of diversified diets are two possible interventions that can help contain anaemia in pregnancy.
